# Gossypiboma with perforation of the umbilicus mimicking a complicated urachal cyst: a case report

**DOI:** 10.1186/s12893-020-00904-7

**Published:** 2020-10-17

**Authors:** Mustapha S. Kabba, Martha Y. Forde, Kevin S. Beckley, Bernadette Johnny, Ann-Marie B. M. Jah-Kabba, Samuel B. Seisay, Alusine M. Dawoh, Temidayo Ogundiran

**Affiliations:** 1 Department of Surgery, College of Medicine and Allied Health Sciences, University of Sierra Leone and Connaught Hospital, University of Sierra Leone Teaching Hospitals Complex, Freetown, Sierra Leone; 2 Department of Radiology, Connaught Hospital, University of Sierra Leone Teaching Hospitals Complex, Freetown, Sierra Leone; 3 College of Medicine, University of Ibadan & University College Hospital, Ibadan, Nigeria

**Keywords:** Case report, Gossypiboma, Intraabdominal abscess, Retained surgical sponge, Urachal cyst

## Abstract

**Background:**

A retained surgical sponge, also known as a gossypiboma, is a rare cause of serious postoperative complications. Diverse retained surgical materials including instruments such as clamps and sutures have been reported, but surgical sponges are the most common material. We report an unusual case of a gossypiboma mimicking a complicated urachal cyst that led to perforation of the umbilicus.

**Case presentation:**

A 38-year-old female patient presented in our facility with a palpable periumbilical mass and discharge of pus from the umbilicus for 7 months after an open appendectomy. Since the onset of symptoms, the patient had been treated conservatively in a peripheral hospital where she had been operated on. As no improvement was seen, an ultrasound scan was performed that suggested an intraperitoneal abscess adjacent to the umbilicus. Consequently, the patient was referred to our specialist outpatient department for surgical intervention. Suspecting a complicated urachal cyst, an exploratory laparotomy was performed but revealed a retained surgical sponge as the underlying cause. The gossypiboma was resected, and the postoperative period was unremarkable.

**Conclusion:**

This case demonstrates that gossypibomas, even though rare, continue to occur. They may clinically and radiologically mimic other pathologies, especially abscesses and tumors. Preventive measures as well as the inclusion of gossypibomas in the differential diagnosis of intraabdominal masses or fistulation detected in patients with a history of surgery are of utmost importance to minimize morbidity, mortality, and potential medicolegal implications.

## Background

A retained surgical sponge, also known as a gossypiboma, is a rare but serious postoperative complication that is a nightmare for every surgeon. A range of retained surgical materials including instruments such as clamps and sutures have been reported, but the surgical sponge, possibly because of its amorphous composition and frequent usage, is the most prevalent material [1, 2]. For reasons such as fear of litigation, the exact incidence of gossypiboma is difficult to ascertain [1]. Silva and Sousa estimated its incidence to be 1:1000–1:1500 laparotomies [3], whereas Bani-Hani et al. estimated it to be much lower (1:5027) [4].

Gossypiboma may lead to chronic abdominal pain, an abdominal mass, bowel obstruction, occult infections, fistulae and transmural migration of the foreign body into the small and large bowel or stomach [1–3]. We report a case of a gossypiboma with spontaneous perforation at the umbilicus mimicking a complicated urachal cyst, which, to the best of our knowledge, has not been reported before.

## Case presentation

**Fig. 1:**
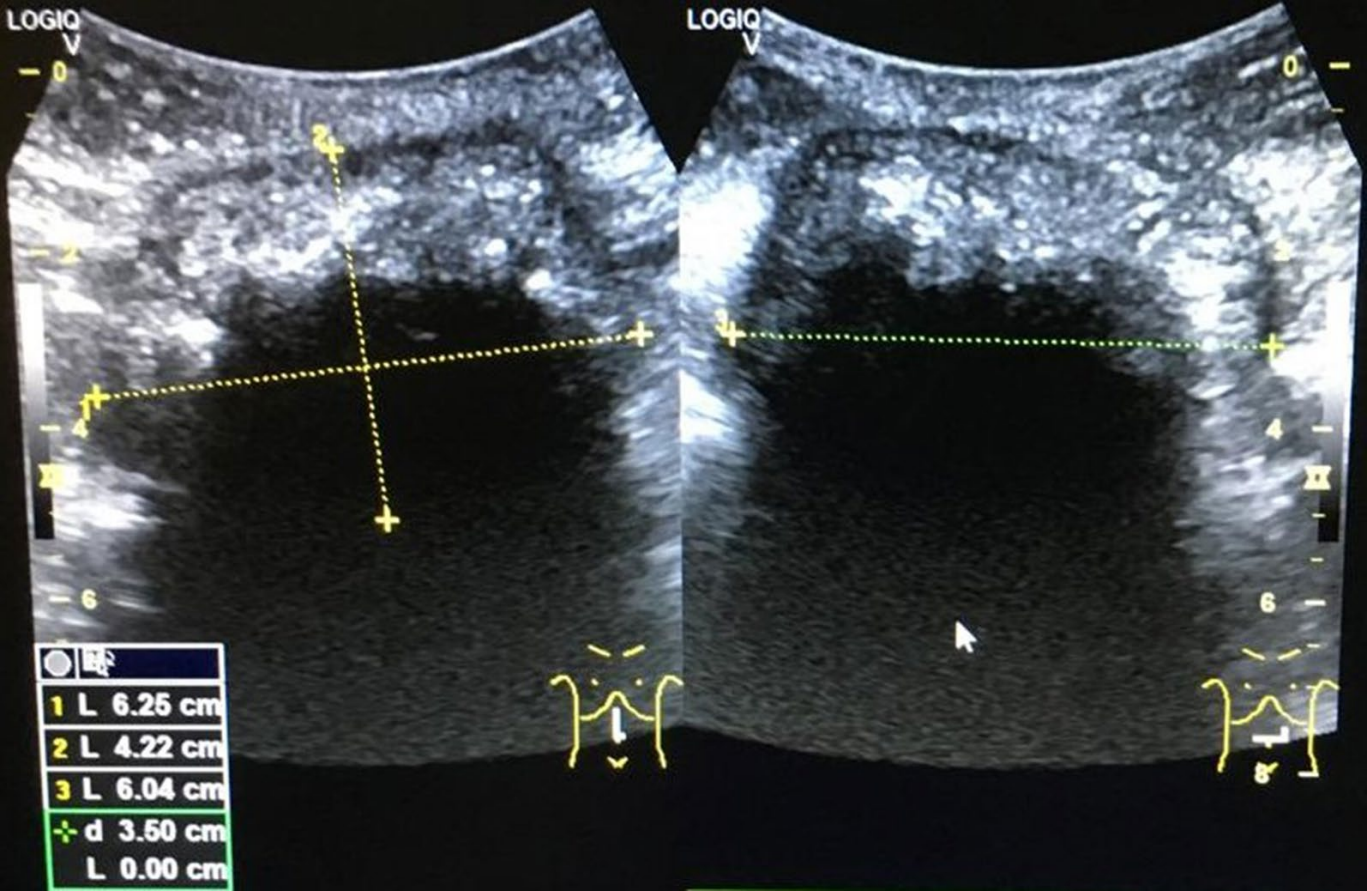
Ultrasound scan showing a well-circumscribed intraabdominal lesion with a complex and irregular internal echo pattern. Strong echoes and posterior acoustic shadowing indicate gas bubbles. Appearances are suspicious of an intraabdominal abscess

A 38-year-old female patient presented to our facility in October 2019 with chronic abdominal pain, swelling in the umbilical region, and pus discharge from the umbilicus for 7 months. The swelling started 3 months after an open appendectomy was performed in a peripheral private hospital using a right paramedian incision. One week after the appearance of swelling, the umbilicus started discharging thick offensive fluid, which was associated with abdominal pain. There was no fever, nausea, or loss of appetite. The patient reported a weight loss of 5 kg in 7 months. Conservative treatment yielded no improvement. As a result of an abdominal ultrasound scan, which suggested an intraperitoneal abscess (Fig. 1), the patient was referred to our specialist outpatient department.

**Fig. 2:**
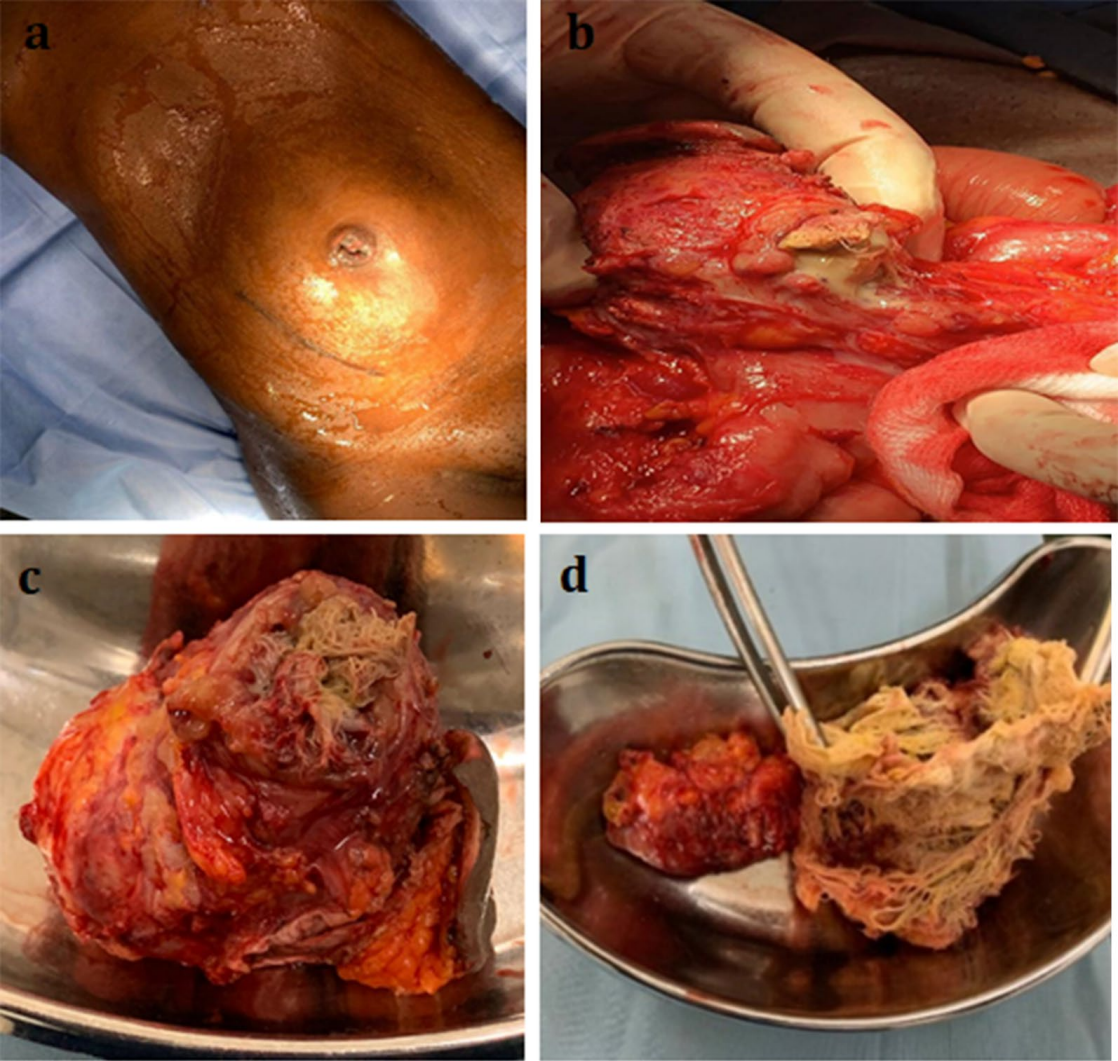
**a** Preoperative image showing periumbilical abdominal distension with an everted umbilicus. **b** Intraoperative image showing the accidentally opened gossypiboma surrounded by bowel loops and adhesions. **c** Resectate including the gossypiboma and the umbilicus. **d** Retained surgical sponge with no identifiable radiopaque marker

Upon presentation, the patient was depressed, dehydrated, and pale. The abdominal examination revealed an everted umbilicus with pus discharge and granulation tissue. In the umbilical region, there was a palpable intraabdominal mass that measured 20 × 16 cm, was moderately tender, and was fixed to the umbilicus but was otherwise mobile (Fig. 2a). There were no signs of peritonitis, and the abdomen was soft with moderate periumbilical distension. The right paramedian appendectomy scar had healed completely and was unremarkable. A routine blood examination showed a hemoglobin concentration of 9.6 g/dL and a white blood cell count of 3.0 × 10^9^/L. Further diagnostic tests and imaging could not be performed due to financial constraints.

Based on the history and physical findings, a provisional diagnosis of an infected urachal cyst or postoperative intraabdominal abscess was made, and the patient was prepared for an exploratory laparotomy.

Intraoperatively, the abdominal cavity was clean. A well-circumscribed 18 × 16 cm adhesive mass was seen around the ileocecal junction. It consisted of an encapsulated lesion at its center, and an ileal segment, the transverse colon, and the umbilicus were all adhered to the mass (Fig. 2b). During adhesiolysis, the wall of the central lesion was accidentally opened, thus surprisingly exposing a surgical sponge and thick offensive pus. The bowel loops were further mobilized, resulting in a large mesenteric defect. Impaired blood supply to the dependent bowel loop necessitated the resection of a 10 cm long ileal loop, and an end-to-end ileo-ileal anastomosis was created. Adhesiolysis and mobilization of the lesion from the transverse colon led to a 3 cm iatrogenic defect, which was sutured. Finally, the encapsulated lesion, including the umbilicus, was completely resected (Fig. 2c). The resectate measured 10 × 8 cm in size and contained a retained surgical sponge (Fig. 2d) within a confined collection of pus.

The postoperative period was uneventful. Bacteriological examinations were not carried out due to financial constraints. Consequently, empiric antibiotic therapy with intravenous ceftriaxone and metronidazole was administered for 5 days and was then switched to oral antibiotics, which were given for an additional 2 days. Early postoperative enteral feeding was well tolerated with normal bowel activity on the third postoperative day. The laparotomy wound healed by primary intention, and the patient was discharged on postoperative day 9. Follow-up visits in our SOP clinic have also been unremarkable.

## Discussion and conclusion

Gossypiboma is described as a retained surgical sponge or towel after a surgical procedure [1, 4]. Other terminologies, such as textiloma, gauzeoma, cottonoid, muslinoma, and cottonballoma, are used to refer to this condition. The most common site of presentation is the abdominal cavity. However, other locations, including the pleural and pericardial cavities, the extremities, the central nervous system, and the breast, have been documented [5]. To the best of our knowledge, a gossypiboma with perforation at the umbilicus has not been reported before.

A retained surgical sponge is a preventable postoperative complication that usually occurs in emergency surgeries, unexpected surgical procedures, prolonged operations, and surgeries on unstable and obese patients. Furthermore, it is associated with inexperienced staff, poor organization in the operating theatre, and rapid or failed sponge counting during an operation [2, 6]. The possible predisposition to the occurrence of the retained foreign body in our patient at her initial surgery could have been an emergency procedure in a poor organizational setting with inadequately trained staff.

Essentially, there are two types of pathological reactions provoked by a retained foreign body: an exudative inflammatory process with the formation of an abscess or an aseptic fibrotic reaction that encapsulates the corpus alienum [6, 7]. In our patient, the gossypiboma led to the formation of an abscess and discharge of its liquid content at one of the weakest points of the abdominal wall, which in this case proved to be the closely related umbilicus—a process equivalent to the commonly observed spontaneous drainage of abscesses via fistula formation.

Intraabdominal gossypibomas are usually reported with signs of an intraabdominal mass, bowel obstruction, intestinal hemorrhage, or fistulation. Rarely, the retained surgical sponge undergoes transmural bowel wall migration without leaving a defect [1, 2, 8].

The diagnosis of gossypiboma is often challenging, as there is generally a wide range of nonspecific manifestations, and most times, the actual diagnosis is not even considered. In line with this phenomenon, neither the peripheral hospital nor our facility included gossypiboma in the differential diagnosis.

The presentation of our patient with a periumbilical mass and discharge of pus at the umbilicus prompted the potential differential diagnosis of an infected urachal cyst. Incomplete urachal obliteration during fetal development gives rise to distinct malformations of the median umbilical ligament, such as a urachal cyst. Most anomalies are asymptomatic and resolve during early infancy, but some go unrecognized until adulthood. These rare cases can present with acute abdominal symptomatology secondary to infection of the urachal remnant [9]. The clinical presentation of urachal cysts is nonspecific. Urachal cysts are mostly asymptomatic until infection occurs. However, a triad of symptoms consisting of a tender midline infraumbilical mass, umbilical discharge, and sepsis should arouse suspicion of an infected urachal remnant, such as a urachal cyst. Without surgical excision, a urachal cyst may slowly increase in size and drain through the umbilicus into the bladder or both, resulting in an alternating sinus [10].

In our patient, a high index of suspicion for gossypiboma should have been raised by the history of surgery associated with persistent abdominal pain, signs of infection, and a palpable mass. A preoperative diagnosis of gossypiboma using the clinical manifestation and recommended imaging modalities was observed by Rajiv et al. in only 75% of cases, highlighting the fact that a significant number of gossypibomas are diagnosed only intraoperatively [11, 12].

In the case of radiopaque impregnation of a foreign body, diagnosis may be facilitated by radiographic and CT images. The sponge we evacuated showed no discernible impregnation, which may have been due to the primary absence of impregnation, as especially seen in resource-poor settings or disintegration [2, 10] of the radiopaque material. In such cases, the foreign object may not be readily identified by imaging.

Removal of the retained foreign body by either open or laparoscopic surgery is the mainstay of treatment. However, if the retained foreign body migrates into the stomach, it could be endoscopically removed [6].

Prevention of gossypibomas is essential and can be achieved by “simply” ensuring and insisting on a thorough pack count before closure of the operating cavity. A routine brief but careful postoperative wound and cavity exploration prior to wound closure should be performed by every surgeon. The use of sponges with radiopaque markers is now strictly recommended. Moreover, newer technologies such as radiofrequency chip identification and barcode scanners are helpful for decreasing the incidence of gossypiboma [13]. However, radiopaque chip identification and barcode scanners are only available in highly developed centers. Theatre staff should be regularly sensitized and trained to avoid this preventable condition.

This case shows that the diagnosis of gossypiboma can be complicated by its nonspecific presentation, which clinically and radiologically imitates other common pathologies, such as abscesses and tumors, and rare conditions, such as urachal cysts. Moreover, gossypiboma is associated with significant morbidity, mortality, and potential medicolegal implications. Consequently, prevention of its occurrence and the inclusion of gossypiboma in the repertoire of actively thought differential diagnoses in patients with intraabdominal masses or fistulation concurrent with a history of surgery are of utmost importance.

## Data Availability

Not applicable.

## Abbreviations

US: Ultrasound Scan
CT: Computed tomography
SOP: Specialist Outpatient Department
